# The sucrose non-fermenting-1-related protein kinases SAPK1 and SAPK2 function collaboratively as positive regulators of salt stress tolerance in rice

**DOI:** 10.1186/s12870-018-1408-0

**Published:** 2018-09-20

**Authors:** Dengji Lou, Houping Wang, Diqiu Yu

**Affiliations:** 1 Key Laboratory of Tropical Plant Resources and Sustainable Use, Xishuangbanna Tropical Botanical Garden, Chinese Academy of Sciences, Kunming, 650223 Yunnan China; 2 University of Chinese Academy of Sciences, Beijing, 100049 China

**Keywords:** Rice, SAPK1, SAPK2, NaCl, CRISPR/Cas9, ROS, Na^+^ toxicity, Osmotic

## Abstract

**Background:**

The sucrose non-fermenting-1-related protein kinase 2 family (SnRK2s) unifies different abiotic stress signals in plants. To date, the functions of two rice SnRK2s, osmotic stress/ABA-activated protein kinase 1 (SAPK1) and SAPK2, have been unknown. We investigated their roles in response to salt stress by generating loss-of-function lines using the CRISPR/Cas9 system and by overexpressing these proteins in transgenic rice plants.

**Results:**

Expression profiling revealed that *SAPK1* and *SAPK2* expression were strongly induced by drought, NaCl, and PEG treatment, but not by ABA. *SAPK2* expression was highest in the leaves, followed by the roots, whereas *SAPK1* was highest expressed in roots followed by leaves. Both proteins were localized to the nucleus and the cytoplasm. Under salt stress, *sapk1, sapk2* and, in particular, *sapk1/2* mutants, exhibited reduced germination rates, more severe growth inhibition, more distinct chlorosis, reduced chlorophyll contents, and reduced survival rates in comparison with the wild-type plants. In contrast, SAPK1- and SAPK2-overexpression lines had increased germination rates and reduced sensitivities to salt; including mild reductions in growth inhibition, reduced chlorosis, increased chlorophyll contents and improved survival rates in comparison with the wild-type plants. These results suggest that SAPK1 and SAPK2 may function collaboratively as positive regulators of salt stress tolerance at the germination and seedling stages. We also found that SAPK1 and SAPK2 affected the osmotic potential following salt stress by promoting the generation of osmotically active metabolites such as proline. SAPK1 and SAPK2 also improved reactive oxygen species (ROS) detoxification following salt stress by promoting the generation of ROS scavengers such as ascorbic acid, and by increasing the expression levels of proteins such as superoxide dismutase (SOD) and catalase (CAT). SAPK1 and SAPK2 may function collaboratively in reducing Na^+^ toxicity by affecting the Na^+^ distribution between roots and shoots, Na^+^ exclusion from the cytoplasm, and Na^+^ sequestration into the vacuoles. These effects may be facilitated through the expression of Na^+^-and K^+^-homeostasis-related genes.

**Conclusion:**

SAPK1 and SAPK2 may function collaboratively as positive regulators of salt stress tolerance at the germination and seedling stages in rice. SAPK1 and SAPK2 may be useful to improve salt tolerance in crop plants.

**Electronic supplementary material:**

The online version of this article (10.1186/s12870-018-1408-0) contains supplementary material, which is available to authorized users.

## Background

Rice (*Oryza sativa*), is one of the most important cereal crops, and is the most sensitive of all cereal crops to salt stress, in particular, at the seedling and reproductive stages [1]. Among the most common environmental stresses, salinity is a major constraint to plant growth and even mild salt stress can result in significant loss of productivity. Plants suffering from high salt stress often have a rapidly occurring osmotic stress and a more slowly forming ionic imbalance [2]. Consequently, molecular damage and arrested growth occur, which ultimately reduce the crop yield.

Salt stress is complex because it implies both ion toxicity and an osmotic component [3]. High salt stress inhibits plant growth through both osmotic stress and ionic stress [4]. Osmotic stress results in inhibition of water uptake, cell elongation, and leaf development. Ionic stress leads to high levels of Na^+^ accumulation in shoots; which decreases protein synthesis, enzymatic reactions and photosynthetic processes [2, 4, 5]. As a result, the production of reactive oxygen species (ROS) is increased, and ultimately the plant growth and biomass is reduced [6]. With salt exposure, plants often display symptoms of Na^+^ toxicity due to Na^+^ accumulation. The main site of Na^+^ toxicity for many plants is the leaves, where photosynthesis and other metabolic processes occur [1, 7]. Therefore, reducing Na^+^ accumulation in the shoots - especially in the leaves - is important to protect plants from ionic Na^+^ stress [8, 9].

To reduce the toxic effects of Na^+^ accumulation, plants have mainly evolved two types of tolerance mechanisms. These are based on either limiting the entry of salt into the roots, or on controlling the salt concentration and distribution [10]. Intracellular Na^+^ is exported out of the cell by the plasma membrane Na^+^/H^+^ antiporters salt overly sensitive (SOSs) pathway [11], or it is sequestered into the vacuole by tonoplast-localized Na^+^/H^+^ or K^+^/H^+^ exchangers (NHXs) [12]. At the tissue level, regulation of Na^+^ loading into the root xylem is essential for limiting Na^+^ accumulation in the shoot [13]. Many of the components related to the regulation of Na^+^ and K^+^ homeostasis in plants have already been characterized; including SOSs, NHXs, and high-affinity K^+^ transporters (HKTs) [13–15].

**Fig. 1:**
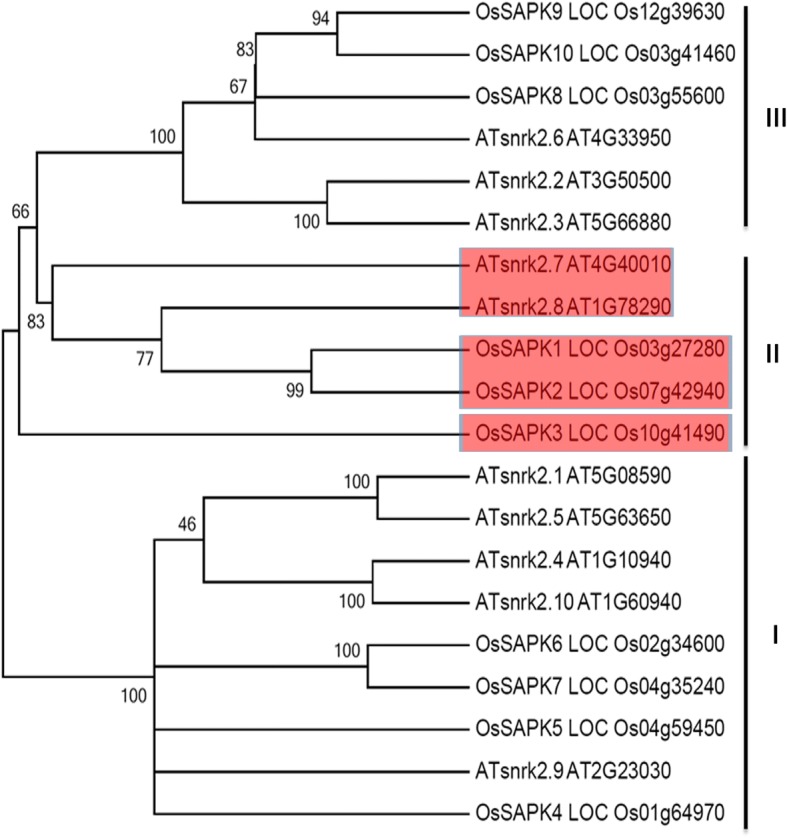
The phylogenetic tree of the SnRK2 family in Arabidopsis and rice. Two closely related protein kinases, OsSAPK1 and OsSAPK2, are involved in subclass II SnRK2 in rice. Subclass II SnRK2s in Arabidopsis and rice are indicated in red

Phosphorylation is one of most important post-translational modifications; affecting activity, localization and stability of target proteins [16]. Under salt stress, protein phosphorylation cascades are activated and play critical roles in rice salt tolerance [17, 18]. The sucrose non-fermenting-1-related protein kinase 2 family (SnRK2s) consists of ten members in both the *Arabidopsis* (SnRK2.1–10) and rice (known as osmotic stress/ABA-activated protein kinases, SAPK1–10) genomes. Based on their domain structures, they can be classified into subclasses I-III (Fig. 1) [17]. Previous studies have confirmed that SnRK2s are involved in abscisic acid (ABA) and stress signaling pathways [19, 20]. Most of the rice and *Arabidopsis* SnRK2 kinases, with the exception of AtSnRK2.9, are activated by salt treatment [17, 21]; suggesting that they may have important roles in plant salt responses. In addition, several *Arabidopsis* (2.2, 2.3, 2.6, 2.7 and 2.8) and rice (SAPK8, SAPK9 and SAPK10) SnRK2 members are activated by ABA [17, 21]. These proteins are key regulators of the ABA signaling pathway; controlling seed development, dormancy and germination, seedling growth and stomata regulation in response to drought [22–24]. The *Arabidopsis* subclass II member SRK2C/SnRK2.8 positively regulates drought tolerance in roots [25], and also positively regulates metabolic processes associated with plant growth [26]. Furthermore, overexpression of *SRK2C/SnRK2.8* enhances drought tolerance [25] and plant growth [26] in *Arabidopsis*. However, Mizoguchi et al. [27] reported that *Arabidopsis snrk2.7*, *snrk2.8*, and *snrk2.7/2.8* mutants do not show any clear differences in survival or visible damage under drought stress in comparison with wild-type plants. In rice, overexpression of *SAPK4* increases salt tolerance by altering the expression levels of ion homeostasis genes [28]. Ectopic expression of *SAPK6* (*OSRK1*) in tobacco reduces ABA sensitivity during seed germination and root elongation [29]. Overexpression of *SAPK6* (*OSRK1*) enhances root growth under salt stress through the phosphorylation of the ABF family in rice [30]. Furthermore, SAPK9 positively regulates ABA-mediated drought stress signaling pathways in rice [31].

Although there has been recent progress investigating the roles of the aforementioned members of SnRK2 subclasses I and III; in rice, our knowledge of the subclass II members is still limited. Previously, we reported that SAPK2, a subclass II member in rice, was strongly activated by drought and salt treatments [32]. We also found that *sapk2* mutants were more sensitive to drought stress than wild-type plants due to the up-regulation of numerous stress-inducible genes [32]. Another member of subclass II, SAPK1, is strongly activated by high osmolarity, in a similar manner to SAPK2 [17]. The kinase domains of SAPK1 and SAPK2 are highly conserved (92% amino acid identity). Therefore, these two subclass II members may regulate some overlapping signal transduction pathways. To date, the roles of SAPK1 and SAPK2 in salt tolerance in rice have not been characterized. In this study, we determined the functions of these proteins in response to salt stress using *sapk1, sapk2* and *sapk1/2* loss-of-function mutants (generated using the CRISPR/Cas9 system) and plants overexpressing these proteins (*SAPK1-OE* and *SAPK2-OE*). We analyzed the expression profiles of SAPK1 and SAPK2 and found that their expression levels were strongly induced by drought, NaCl, and PEG treatment, but not by ABA. In addition, *SAPK2* expression was highest in the leaves, followed by the roots; whereas *SAPK1* expression was highest in the roots, followed by the leaves. The localization patterns of *SAPK1* and *SAPK2* were similar in the cytoplasm and nucleus. Under salt stress, *sapk1, sapk2* and *sapk1/2* mutants (especially *sapk1/2* mutants) exhibited reduced germination rates, severe growth inhibition, more advanced chlorosis, reduced chlorophyll contents, and reduced survival rates in comparison with the wild type. In contrast, *SAPK1-OE* and *SAPK2-OE* lines had increased germination rates, reduced salt sensitivities (including milder growth inhibition), increased chlorophyll contents, and increased survival rates in comparison with the wild type. Finally, we analyzed the effects of SAPK1 and SAPK2 on osmotic stress, ROS detoxification and Na^+^, K^+^ accumulation. Together, our results suggest that SAPK1 and SAPK2 may function collaboratively as positive regulators of salt stress tolerance at the germination and seedling stages in rice.

## Results

### Expression profiles and subcellular localization analyses of *SAPK1* and *SAPK2* in rice

SAPK1 and SAPK2 are rice SnRK2 II subfamily members. The open reading frame of SAPK1 consists of 1,029 bp and encodes a 342-amino acid polypeptide (LOC_Os03g27280). The open reading frame of SAPK2 consists of 1,020 bp and encodes a 339-amino acid polypeptide (LOC_Os07g42940). In our previous study, we showed that *SAPK2* is induced by drought, NaCl, and PEG [32]. To further clarify the roles of *SAPK1* and *SAPK2* in rice, we examined their expression profiles more precisely.

**Fig. 2:**
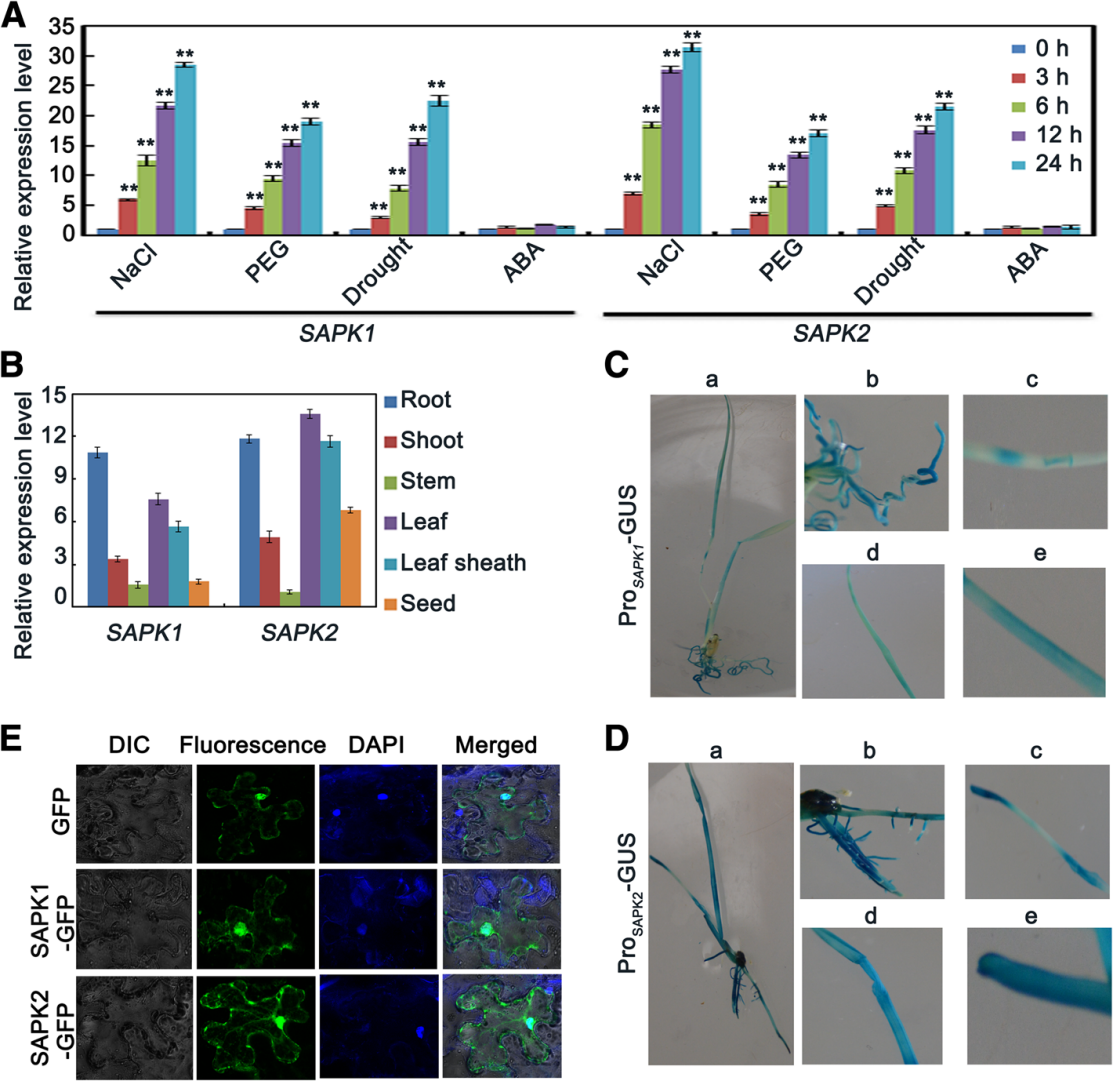
Analyses of *SAPK1* and *SAPK2* expression levels and locations. (**a**) Analysis of *SAPK1* and *SAPK2* expression levels under NaCl, PEG, drought and ABA treatments. The expression level was assessed by qRT-PCR. The expression level is indicated as a relative value, and expression at o hour was defined as 1.0. Error bars indicate the SD (*n* = 3). ** indicate statistically significant differences between 0 h and other times (*P* < 0.01). (**b**) qRT–PCR analysis of *SAPK1* and *SAPK2* expression in different tissues: root, shoot, stem, leaf, leaf sheath and seed. The expression level is indicated as a relative value, and expression in stem was defined as 1.0. Error bars indicate the SD (n = 3). (**c**) β–Glucuronidase gene (GUS) staining of Pro_*SAPK1*_-GUS and (**d**) Pro_*SAPK2*_-GUS transgenic plants in 2-week-old seedlings. Each panel shows whole plants (**a**) root (**b**) stem (**c**) leaf and (**d**) leaf sheath after GUS staining. (**e**) Subcellular localization of *SAPK1* and *SAPK2*–green fluorescent protein (GFP) fusion proteins in protoplasts. Protoplasts from Nicotiana benthamiana leaves were transiently transfected and incubated 48 h

First, we measured the induced expression of *SAPK1* and *SAPK2* in response to certain abiotic stresses using quantitative reverse transcription-PCR (qRT-PCR). *SAPK1* and *SAPK2* expression were both strongly up-regulated by drought, NaCl, and PEG treatment; but not by ABA treatment (Fig. 2A). These results show that *SAPK1* and *SAPK2* both respond to salt treatment and may be involved in responses to salinity stress.

Next, we compared the *SAPK1* and *SAPK2* expression patterns in various tissues. To do this, we used qRT-PCR, and histochemical analysis of transgenic plants expressing the β-glucuronidase (GUS) gene driven by their own promoters. *SAPK2* expression was highest in the leaves, followed by the roots; which is consistent with a previous study [32]. In contrast, *SAPK1* expression was highest in the roots, followed by the leaves (Fig. 2B). Histochemical analysis revealed the strongest *SAPK2* expression was in the leaves, while the strongest *SAPK1* expression was in the roots (Fig. 2C and D). We also examined the subcellular localization of SAPK1 and SAPK2 using fusions to green fluorescent protein (GFP) in tobacco mesophyll protoplasts (Fig. 2E). The localization patterns were similar in the cytoplasm and nucleus, and were analogous to those of the *Arabidopsis* subclass II SnRK2s SRK2C and SRK2F [27]. Together, these results suggest that *SAPK1* and *SAPK2* have different tissue specificities, but they might be functionally collaboratively at the cellular level.

**Fig. 3:**
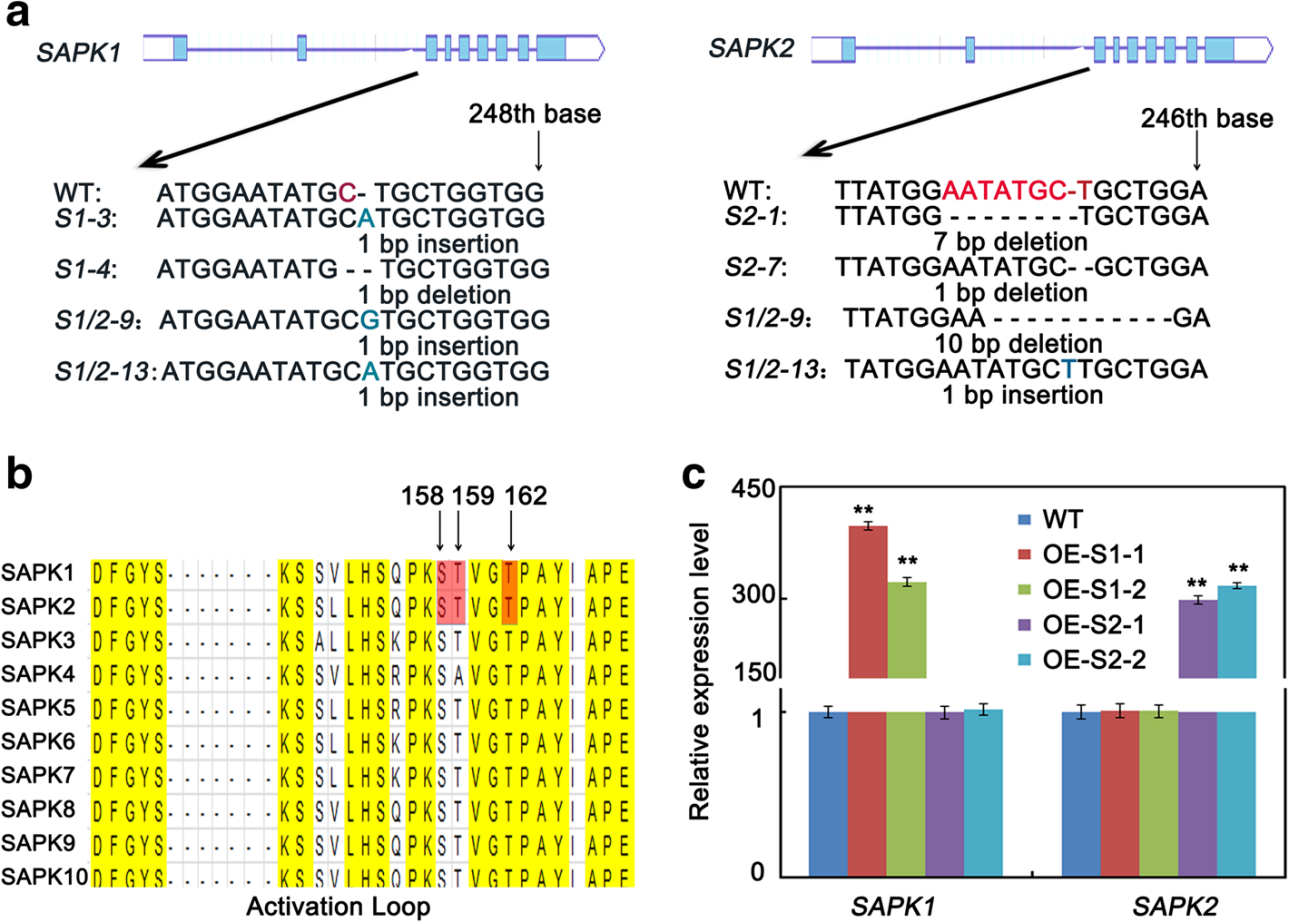
The detection of *sapk1, sapk2 and sapk1/2* mutants and overexpression plants of SAPK1 and SAPK2. (**a**) Diagram showing that sgRNAs targets of *SAPK1* and *SAPK2* were used in the CRISPR/Cas9 system to generate *sapk1, sapk2 and sapk1/2* mutants. Two independent mutant lines *of sapk1, sapk2 and sapk1/2* with different deletions were obtained. (**b**) Ser and Thr residues in the Activation Loop are essential for the activity and/or activation of SAPK1 and SAPK2 in Response to Hyperosmotic Stress. (**c**). Accumulation of *SAPK1* and *SAPK2* transcripts in SAPK1-OE lines, SAPK2-OE lines and wide type plants. Transcript accumulation was assessed by qRT-PCR. Error bars indicate the SD (*n* = 3). ** indicate statistically significant differences between mutant lines and wild type plants (*P* < 0.01)

To further investigate the functions of *SAPK1* and *SAPK2* (Fig. 1a), we established *sapk1* single mutants *(sapk1–3* and *sapk1–4)*; *sapk2* single mutants *(sapk2–1* and *sapk2–7)* and *sapk1/2* double mutants (*sapk1/2–9* and *sapk 1/2–13)* using the CRISPR/Cas9 system. Kobayashi et al. [17] previously reported that Ser-158, Thr-159, and Thr-162 are essential for SAPK1 activity or activation (Fig. 3b). Because the kinase domains of SAPK1 and SAPK2 are highly conserved (92% amino acid identity), we predicted that Ser-158, Thr-159, and Thr-162 are also essential for SAPK2 activity or activation (Fig. 3b). The third coding exons of *SAPK1* and *SAPK2* were selected for guide RNA design. We determined that the *SAPK1* and *SAPK2* coding nucleotide sequences had frame shift mutations in the *sapk1* and *sapk2* single mutants and in the *sapk1/2* double mutants. These mutations resulted in premature termination of the SAPK1 and SAPK2 amino acid sequences before Ser-158 (Fig. 3a). These results confirmed that the *sapk1* and *sapk2* single mutants and the *sapk1/2* double mutants were non-functional.

### SAPK1 and SAPK2 function in responses to salt stress at the germination and post germination stages

**Fig. 4:**
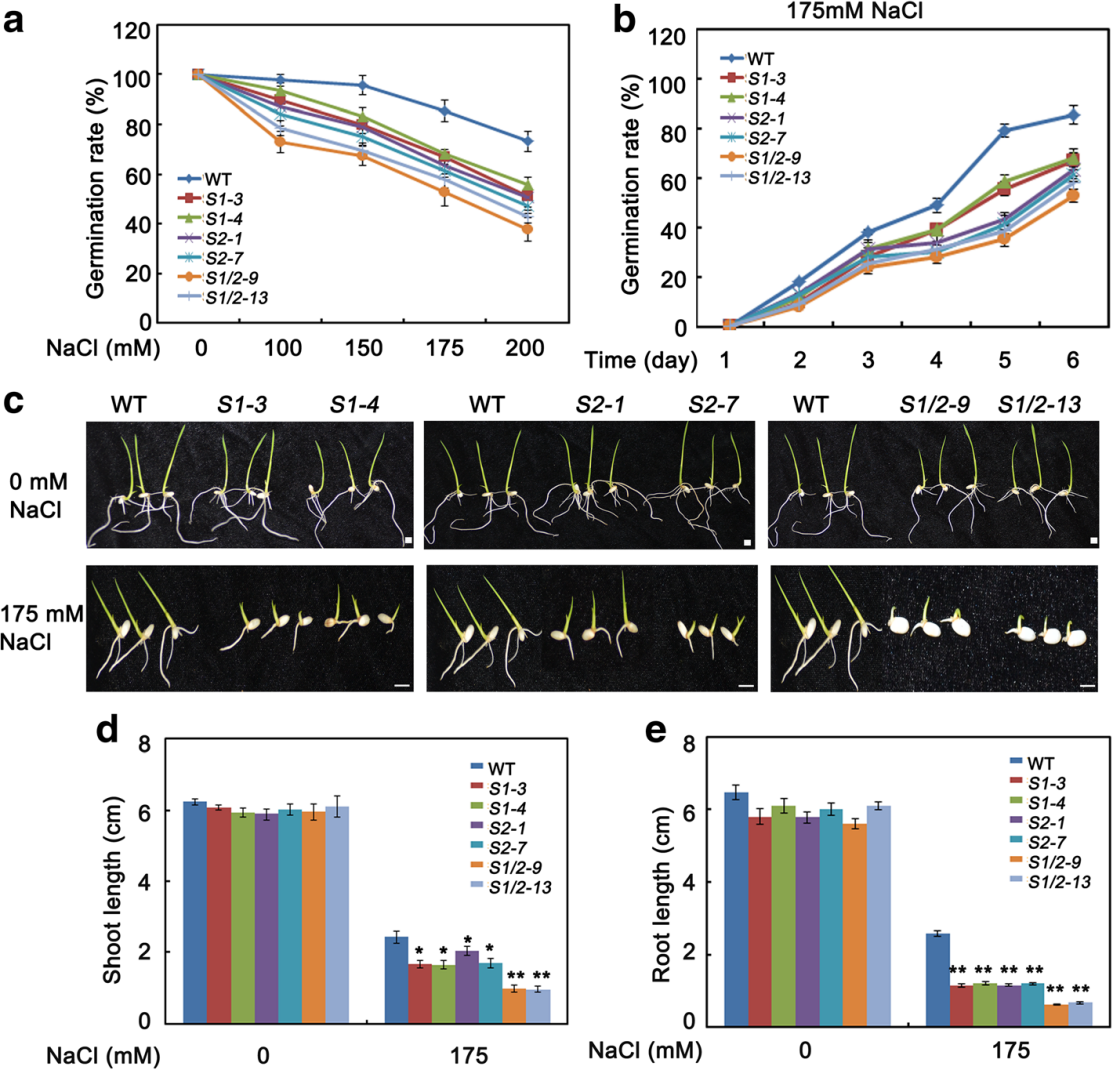
Phenotypic characterization of *sapk1, sapk2 and sapk1/2* mutants at germination and post germination stage. (**a**) Seed germination analyses: the germination of wild-type (WT) and *sapk1, sapk2, sapk1/2* mutants’ seeds on day 6 after stratification on medium supplemented with different concentrations of NaCl. (**b**) The germination rates of WT and *sapk1, sapk2 and sapk1/2* mutants’ seeds on 175 mM NaCl medium over 6 days. All experiments were performed three times each, evaluating more than 60 seeds. (**c**) Early seedling development analyses: WT and *sapk1, sapk2, sapk1/2* mutants’ seeds were geminated in MS medium, and after 3 days all were transferred to 175 mM NaCl-containing medium in a vertical manner. The pictures of representative seedlings were taken 7 days after transfer. (**d**) Shoot and (**e**) Root elongation analyses corresponding to (**c**). Error bars indicate the SD (*n* = 3). * indicate statistically significant differences between mutant lines and wild type plants (*P* < 0.05). ** indicate statistically significant differences between mutant lines and wild type plants (*P* < 0.01)

Seed germination is the most important phase during seedling establishment. However, salt stress is one of the most important factors limiting seed germination. To clarify the roles of SAPK1 and SAPK2 in salinity stress responses, we first compared the germination rate between the *sapk1*, *sapk2* and *sapk1/2* mutants and the wild type on an NaCl-containing medium. Under normal conditions, there were no significant differences in the germination rates between the mutants and the wild type (Fig. 4a). We then germinated the *sapk1*, *sapk2*, *sapk1/2* and wild-type seeds on half-strength Murashige and Skoog (MS) agar medium supplemented with various concentrations of NaCl. The germination rates were calculated based on radicle emergence. As shown in Fig. 4a, on high-salt media (150, 175 or 200 mM NaCl), the germination of *sapk1*, *sapk2* and *sapk1/2* seeds were significantly inhibited in comparison with that of wild-type seeds. With 175 mM NaCl, approximately 34%, 39% and 48% of *sapk1*, *sapk2* and *sapk1/2* seeds, respectively, failed to germinate. These rates were significantly higher than that of the wild-type seeds (approximately 15%; Fig. 4b). These observations indicate that the *sapk1*, *sapk2* and *sapk1/2* mutants are more sensitive to salt stress than wild type during germination. Furthermore, they suggest that SAPK1 and SAPK2 are required for germination under salt stress, and may function collaboratively.

Next, we evaluated the post-germination development of *sapk1*, *sapk2* and *sapk1/2* mutants and wild-type seedlings. As shown in Fig. 4(c, d and e), there were no significant difference in the shoot and root lengths between the mutants and the wild type without salt treatment. However, with 175 mM NaCl treatment, the shoot lengths of the *sapk1* and *sapk2* mutants (1.60–1.70 cm and 1.70–1.90 cm, respectively) were slightly shorter than those of the wild type (2.43 cm). Furthermore, the shoot lengths of the *sapk1/*2 double mutants (0.98 cm) were shorter in comparison with the *sapk1* and *sapk2* single mutants (Fig. 4c and d). In a root growth assay, the *sapk1*, *sapk2* and *sapk1/*2 mutants were all more sensitive to salt treatment in comparison with the wild-type plants (Fig. 4c and e). The root lengths of the *sapk1/*2 double mutants (0.62 cm) were 50% of those of the *sapk1* and *sapk2* mutants (1.16 cm and 1.18 cm, respectively) and were 24% of the wild-type plants (2.59 cm) (Fig. 4c and e). Taken together, these results suggest that SAPK1 and SAPK2 have positive regulatory roles in salt stress responses and their functions are collaborative at the post-germination development stage.

### SAPK1- and SAPK2-overexpression lines are less sensitive to salt stress at the germination and post-germination stages

To further assess the roles of SAPK1 and SAPK2 in salt stress responses at the germination and post-germination stages, we generated transgenic rice expressing SAPK1 and SAPK2 under the control of the 35S promoter (*OE-SAPK1–1*, *OE-SAPK1–2*, *OE-SAPK2–1*, *OE-SAPK2–2*). We analyzed the expression levels of *SAPK1* and *SAPK2* in transgenic rice lines using qRT-PCR. We found that the expression levels of *SAPK1* and *SAPK2* in the *SAPK1-OE* and *SAPK2-OE* lines, respectively, were significantly higher than those of wild-type plants (Fig. 3c). Next, we analyzed the phenotypes of the *SAPK1-OE* and *SAPK2-OE* lines under salt stress. Without NaCl treatment, there were no significant differences in the germination rates and shoot and root lengths between the *SAPK1-OE* and *SAPK2-OE* lines and the wild type.

**Fig. 5:**
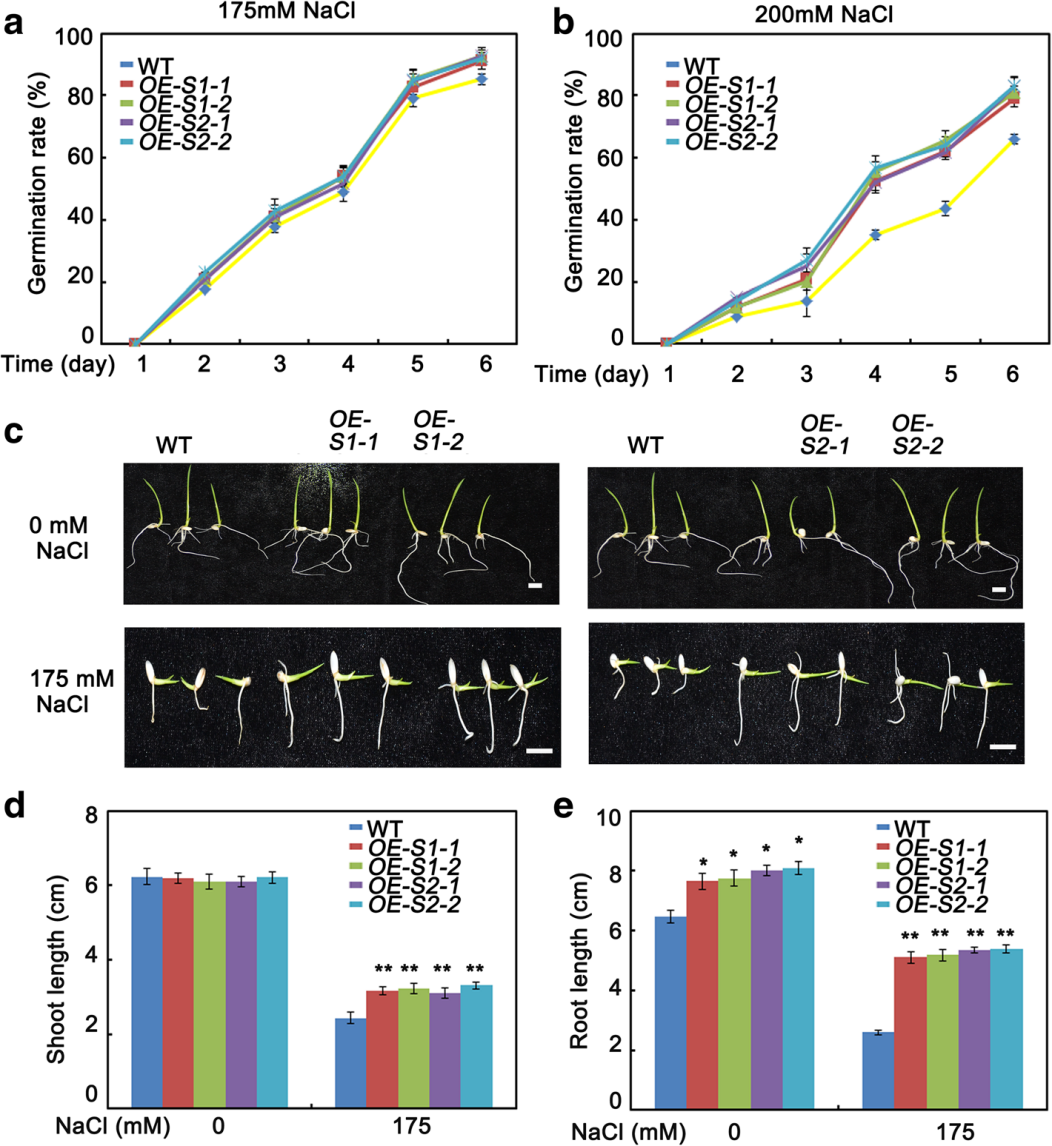
Phenotypic characterization of SAPK1-OE and SAPK2-OE plants at germination and post germination stage. (**a**) Seed germination analyses: the germination of wild-type (WT), SAPK1-OE and SAPK2-OE plants’ seeds on day 6 after stratification on medium supplemented with different concentrations of NaCl. (**b**) The germination rates of WT, SAPK1-OE and SAPK2-OE plants’ seeds on 175 mM NaCl medium over 6 days. All experiments were performed three times each, evaluating more than 60 seeds. (**c**) Early seedling development analyses: WT, SAPK1-OE and SAPK2-OE plants’ seeds were geminated in MS medium, and after 3 days all were transferred to 175 mM NaCl-containing medium in a vertical manner. The pictures of representative seedlings were taken 7 days after transfer. (**d**) Shoot and (**e**) Root elongation analyses corresponding to **c**. Error bars indicate the SD (n = 3). * indicate statistically significant differences between overexpression lines and wild type plants (P < 0.05). ** indicate statistically significant differences between overexpression lines and wild type plants (P < 0.01)

With 175 mM NaCl treatment, the germination rates of the *SAPK1-OE* and *SAPK2-OE* lines were significantly higher than that of the wild type (Fig. 5a and b). With 200 mM NaCl, 20% and 18% of the *SAPK1-OE* and *SAPK2-OE* lines, respectively, failed to germinate. These rates were significantly lower than that of the wild type (44%; Fig. 5b). In a post-germination growth assay, the *SAPK1-OE* and *SAPK2-OE* lines exhibited less sensitivity to salt stress than the wild-type plants. The combined shoot and root lengths of the *SAPK1-OE* and *SAPK2-OE* lines (5.14 cm and 5.42 cm, respectively) were much longer than that of the wild-type plants (2.58 cm) (Fig. 5c-e). Together, these results further support the hypothesis that SAPK1 and SAPK2 have positive regulatory roles in salt stress responses at the germination and post-germination development stages.

### SAPK1 and SAPK2 positively regulate salt tolerance at the seedling stage

**Fig. 6:**
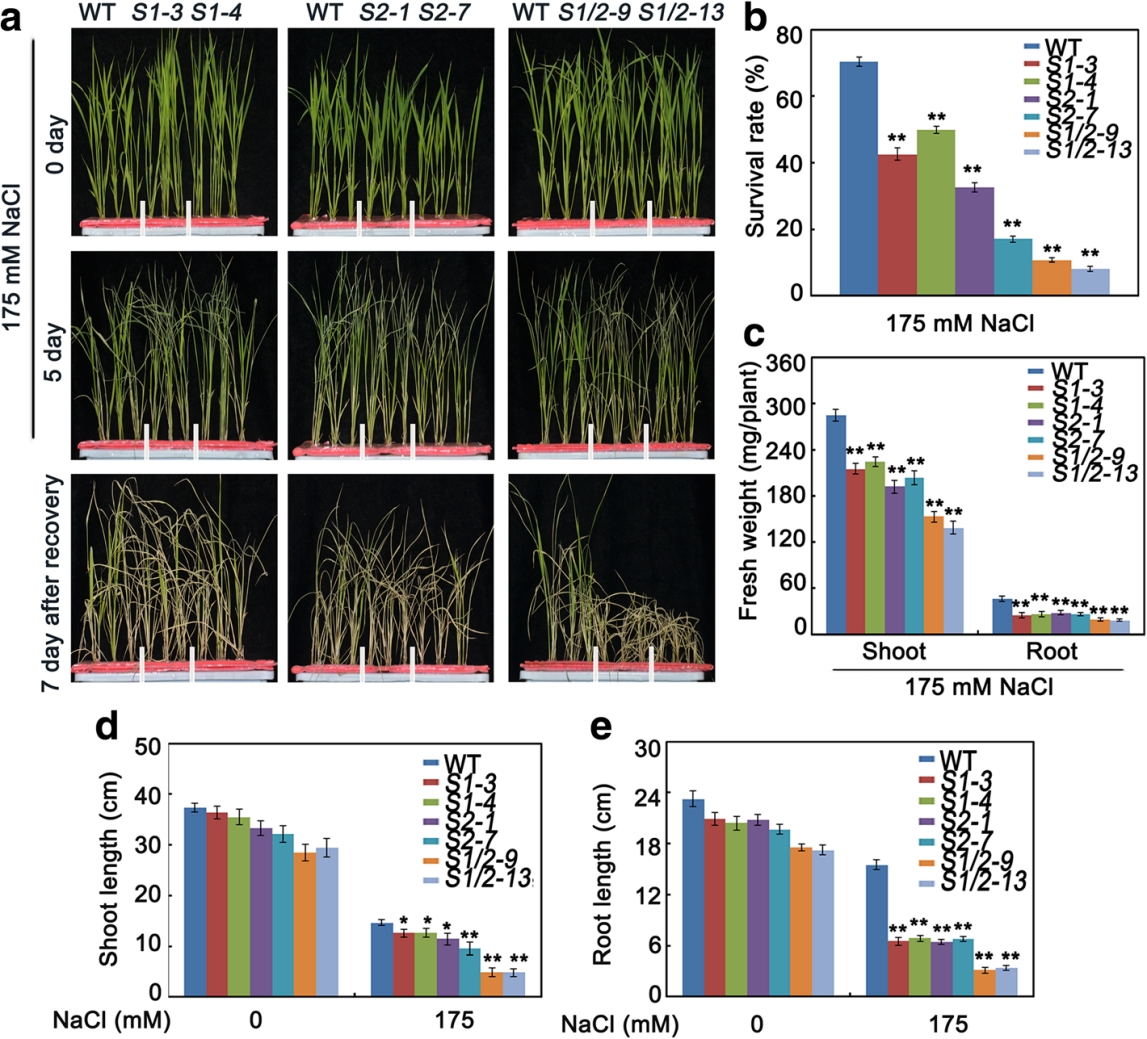
Salt stress tolerance assays of sapk1, sapk2 and sapk1/2 mutants. (**a**) Phenotype of *sapk1, sapk2 and sapk1/2* mutants and wild-type plants before salt stress, after salt stress and re-watered for 7 days. Salt stress analysis was repeated three times. In each repeated experiment, at least 40 plants were used for each individual line. One representative picture was shown. (**b**) Survival rates corresponding to **a**. (**c**) Fresh weight analyses of *sapk1, sapk2, sapk1/2* mutants and wild-type plants under salt stress conditions. (**d**) Shoot and (**e**) Root elongation analyses corresponding to **a**. Error bars indicate the SD (n = 3). * indicate statistically significant differences between mutant lines and wild type plants (P < 0.05). ** indicate statistically significant differences between mutant lines and wild type plants (P < 0.01)

Salt stress usually inhibits plant growth at the seedling stage. To evaluate the physiological responses of *sapk1*, *sapk2* and *sapk1/*2 mutants after salt stress, 4-week-old plants were exposed to half-strength liquid MS medium supplemented with 175 mM NaCl for 5 d. The survival rate was calculated after plants were allowed to recover in fresh half-strength liquid MS medium for 7 d. The survival rate of the *sapk1/*2double mutants (10%) was only 30% of those of *sapk1* and *sapk2* (30% and 40%, respectively) and was only 14% of that of the wild-type plants (70%; Fig. 6a and b).

After treatment with NaCl for 5 d, salt stress caused more visible damage to *sapk1*, *sapk2* and *sapk1/2* mutants than to the wild-type plants, although both plants grew similarly without salt stress (Fig. 6a). Subsequent comparisons of the fresh weights and the lengths of shoots and roots between the mutants and the wild-type plants showed that the NaCl treatment substantially suppressed the growth of all the tissues tested (Fig. 6c, d and e). In the *sapk*1 and *sapk2* mutants, the shoot fresh weights and shoot lengths were only 70% and 80% of those of the wild-type plants, respectively (Fig. 6c and d). However, the most severe damage was found in the roots, with the root fresh weights and root lengths about 60% and 40% of those of the wild-type plants, respectively (Fig. 6c and e). In the *sapk1/2* mutants, shoot and root growth were severely affected. For example, the fresh weights of the shoot and the root were only 48% and 42% of those of the wild-type plants, respectively (Fig. 6c); and the lengths of the shoots and roots was 33% and 21% of those of the wild-type plants, respectively (Fig. 6d and e).

Salt stress causes a significant reduction in the abundance of photosynthetic pigments in plants. The chlorophyll contents of ten seedlings exposed to salt stress were analyzed. Following salt treatment, the chlorophyll contents were greatly reduced in all the plants; however, the chlorophyll contents were most substantially reduced in the *sapk1*, *sapk2* and *sapk1/2* mutants than in the wild-type plants (Fig. 8a). This result follows a similar pattern to that found for survival rates. These results show that the mutations in *sapk1*, *sapk2* and *sapk1/2* obviously affect growth and salt tolerance at the seedling growth stage.

**Fig. 7:**
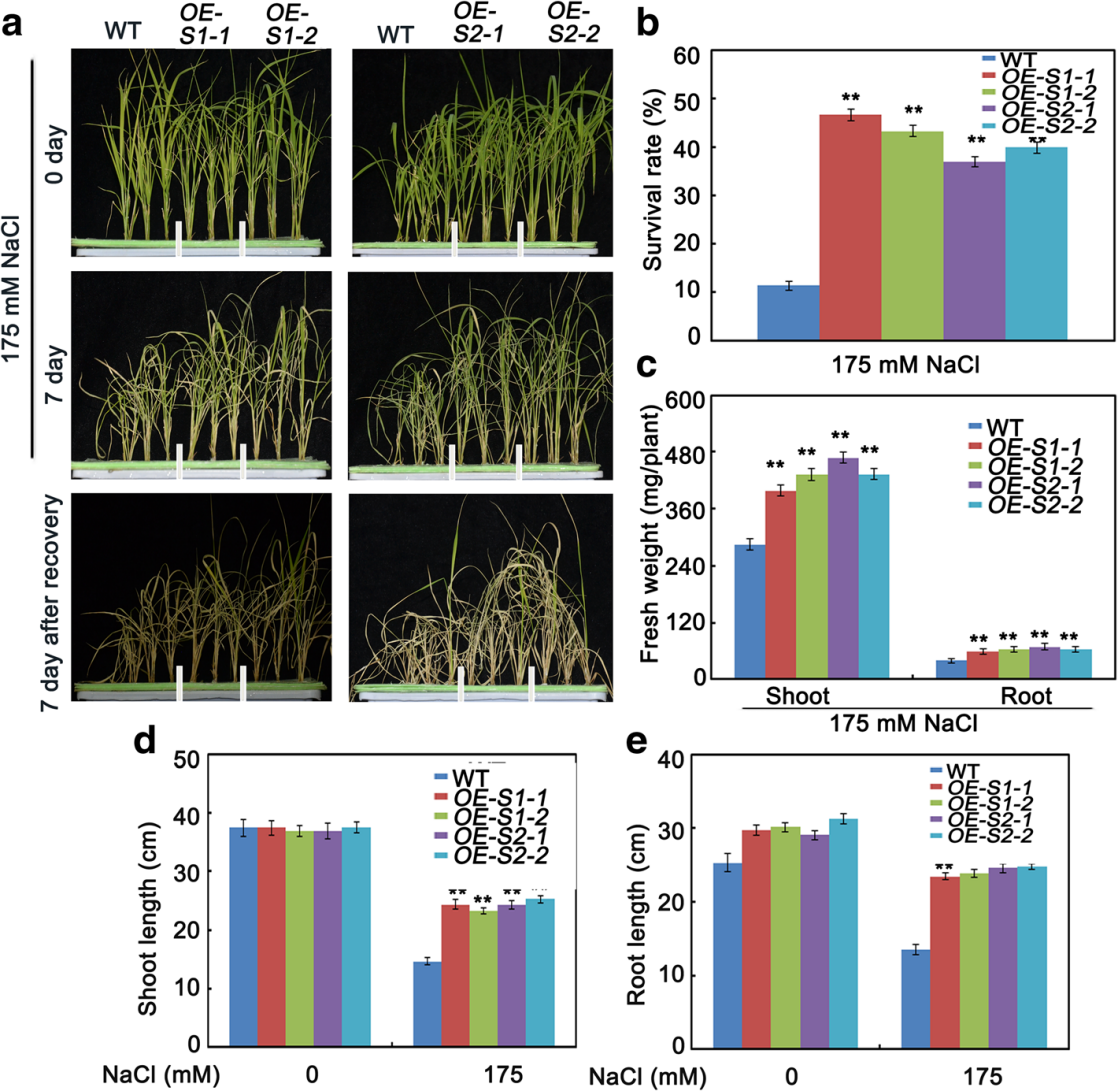
Salt stress tolerance assays of SAPK1-OE and SAPK2-OE plants. (**a**) Phenotype of SAPK1-OE, SAPK2-OE plants and wild-type plants before salt stress, after salt stress and re-watered for 7 days. Salt stress analysis was repeated three times. In each repeated experiment, at least 40 plants were used for each individual line. One representative picture was shown. (**b**) Survival rates corresponding to **a**. (**c**) Fresh weight analyses of SAPK1-OE, SAPK2-OE plants and wild-type plants under salt stress conditions. (**d**) Shoot and (**e**) Root elongation analyses corresponding to **c**. Error bars indicate the SD (n = 3). ** indicate statistically significant differences between overexpression lines and wild type plants (P < 0.01)

**Fig. 8:**
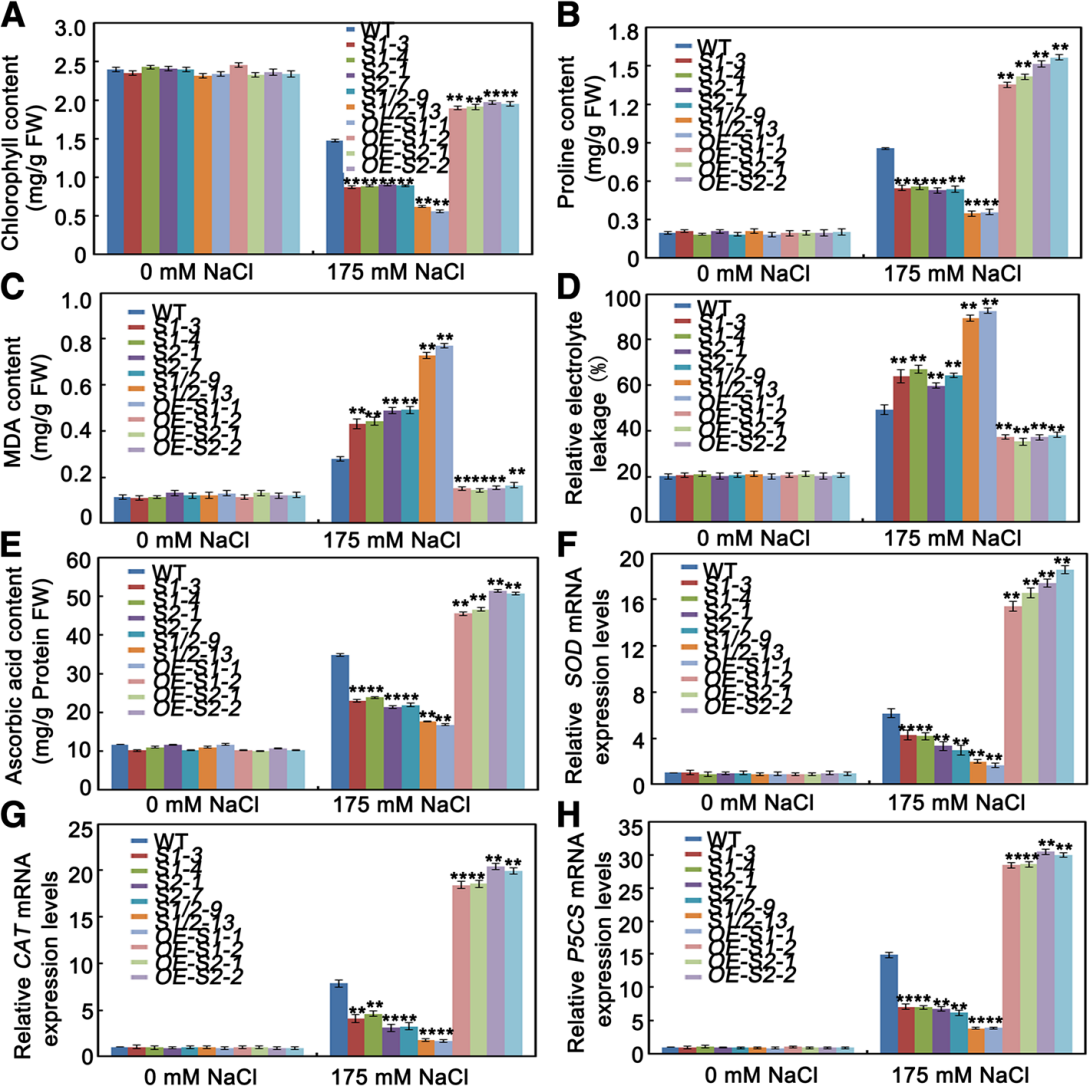
Analysis contents of chlorophyll, proline, MDA and ascorbic acid, relative electrolyte leakage and relative expression levels of *SOD, CAT*, *P5CS.* (**a**) Chlorophyll content (**b**) proline content (**c**) MDA content (**d**) relative electrolyte leakage (**e**) ascorbic acid content in leaf tissues sampled from *sapk1, sapk2 and sapk1/2* mutants; SAPK1-OE, SAPK2-OE plants and wild-type plants before and after salt stress. (**f**) Transcript level of *SOD* (**g**) *CAT* and (**h**) *P5CS* in *sapk1, sapk2 and sapk1/2* mutants; SAPK1-OE, SAPK2-OE plants and wild-type plants leaves before and after salt stress. Error bars indicate the SD (n = 3). ** indicate statistically significant differences between mutant lines and wild type plants (P < 0.01)

To further confirm the roles of SAPK1 and SAPK2 in salt stress responses, we treated *SAPK1-OE* and *SAPK2-OE* transgenic plants with 175 mM NaCl. Four-week-old *SAPK1-OE* or *SAPK2-OE* plants were exposed to half-strength liquid MS medium supplemented with 175 mM NaCl for 7 d. The *SAPK1-OE* and *SAPK2-OE* plants had reduced sensitivity to salt stress, and had milder growth inhibition and chlorosis than the wild-type plants. The transgenic plants also had increased fresh weights, longer shoot and root lengths and higher chlorophyll contents (Fig. 7a, c, d, e and Fig. 8a). The survival rates of *SAPK1-OE* (about 36%) and *SAPK2-OE* (about 46%) were much higher than those of the wild-type plants (11%; Fig. 7a and b). Collectively, these observations further support the hypothesis that SAPK1 and SAPK2 function collaboratively as positive regulators of salt stress tolerance at the rice seedling stage.

### SAPK1 and SAPK2 involved in salt stress response through osmotic adjustment at seedling stage

Accumulated free proline can stabilize subcellular structures and facilitate cell recovery from damage caused by abiotic stress [33]. We compared the proline contents of the *sapk1*, *sapk2* and *sapk1/*2 mutants, the *SAPK1-OE* and *SAPK2-OE* lines and the wild-type plants. No significant difference in proline contents were detected between each of the plant lines under normal conditions. However, under salt stress, the proline content was reduced in the *sapk1, sapk2* and *sapk1/2* mutants (especially in the *sapk1/2* mutants) and was much higher in the *SAPK1-OE* and *SAPK2-OE* lines than in the wild-type plants (Fig. 8b).

Proline biosynthesis is catalyzed by OsP5CS. According to our qRT-PCR data, the *OsP5CS* expression level was much lower in the *sapk1, sapk2* and *sapk1/2* mutants (especially in the *sapk1/2* mutants) and was much higher in the *SAPK1-OE* and *SAPK2-OE* lines than in wild-type plants under salt stress conditions (Fig. 8h).

Taken together, these results suggest that SAPK1 and SAPK2 regulate salt stress responses by affecting the accumulation of compatible solutes such as proline.

### SAPK1 and SAPK2 involved in salt stress response through ROS detoxification at seedling stage

To investigate the degree of membrane peroxidation, we measured the relative electrolyte leakage and malondialdehyde (MDA) content under either normal or 175 mM NaCl conditions. Without NaCl treatment, there were no differences in the relative electrolyte leakage and MDA contents between the *sapk1, sapk2* and *sapk1/2* mutants, the *SAPK1-OE* and *SAPK2-OE* lines and the wild-type plants (Fig. 8c and d). With 175 mM NaCl, the relative electrolyte leakage and MDA contents in the *sapk1*, *sapk2* and *sapk1/*2 mutants were significantly higher than those of the wild-type plants. Moreover, the measurements from the *sapk1/*2 double mutants were much higher than those from the *sapk1* and *sapk2* single mutants (Fig. 8c and d). In contrast, the relative electrolyte leakage and MDA contents were significantly lower in the *SAPK1-OE* and *SAPK2-OE* lines than in the wild-type plants (Fig. 8c and d).

Because MDA is the product of lipid peroxidation arising from ROS activity [34], we speculated that SAPK1 and SAPK2 affect salt tolerance via ROS detoxification. To investigate this hypothesis, we compared the ascorbic acid contents and the expression levels of superoxide dismutase (SOD) and catalase (CAT) between the *sapk1, sapk2* and *sapk1/2* mutants, the *SAPK1-OE* and *SAPK2-OE* lines, and the wild-type plants. As shown in Fig. 8(e–g), in the absence of salt treatment there were no significant differences in the ascorbic acid contents and SOD and CAT expression levels in any of the lines. With salt treatment, the ascorbic acid contents and SOD and CAT expression levels were significantly reduced in the *sapk1, sapk2* and *sapk1/2* mutants (especially in the *sapk1/2* mutants) in comparison with the wild-type plants. In contrast, these parameters were significantly increased in the *SAPK1-OE* and *SAPK2-OE* lines.

These results show that SAPK1 and SAPK2 function collaboratively in their ROS scavenging activity in response to salt stress at the seedling stage in rice.

### SAPK1 and SAPK2 affect Na^+^/K^+^ homeostasis under salt stress

**Fig. 9:**
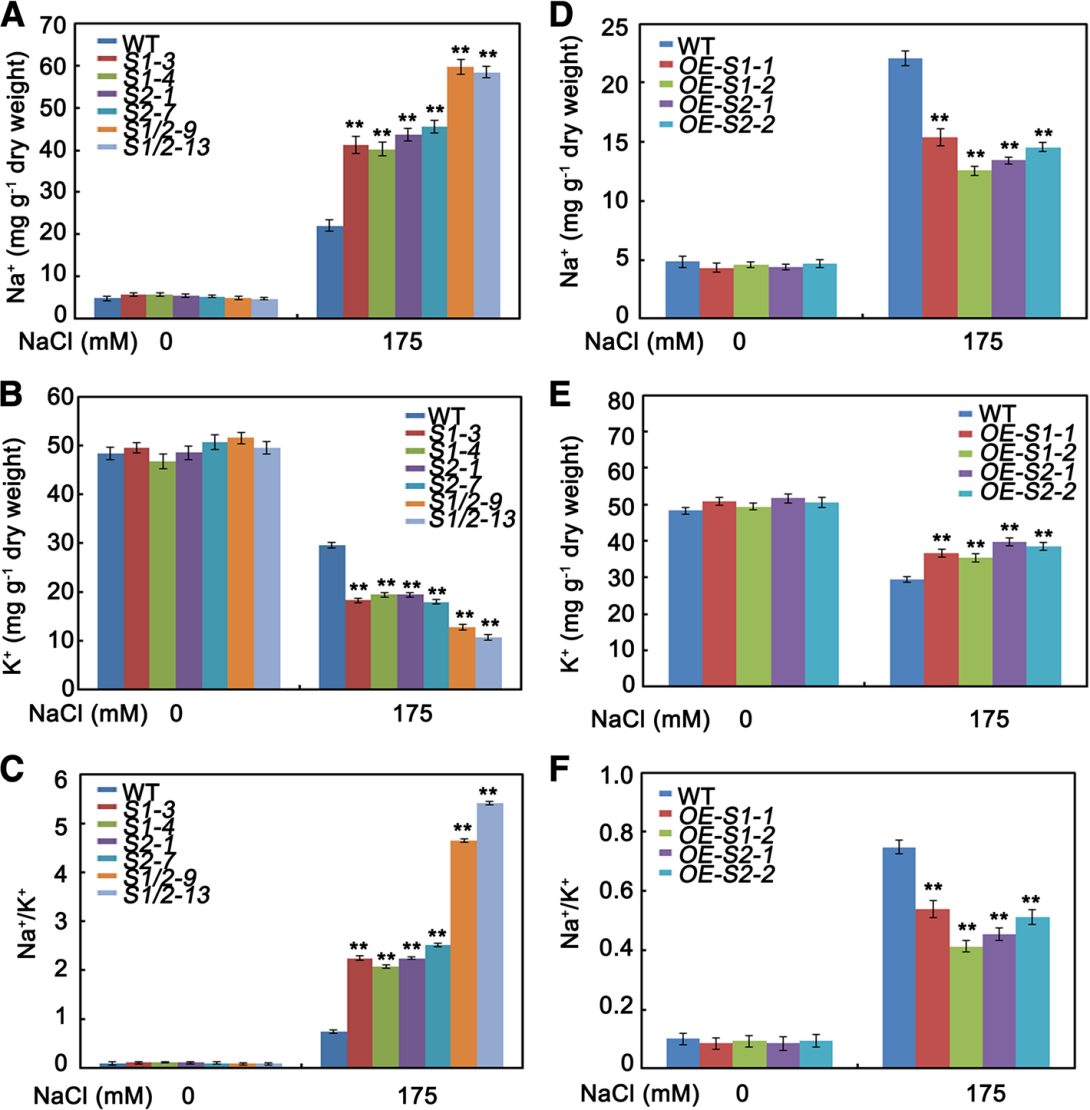
Na^+^/K^+^ homeostasis in *sapk1, sapk2 and sapk1/2* mutants; SAPK1-OE, SAPK2-OE plants and wild-type plants. (**a**) and (**d**)Na^+^ contents: 4-week-old plants were treated with 0 mM or 175 mM NaCl and samples were harvested 5 days later. (**b**) and (**e**) K^+^ contents: 4-week-old plants were treated with 0 mM or 175 mM NaCl and samples were harvested 5 days later. (**c**) and (**f**) Na^+^/K^+^ ratio. Values shown are means from three independent experiments. Error bars indicate the SD (n = 3). ** indicate statistically significant differences between mutant lines and wild type plants (P < 0.01)

**Fig. 10:**
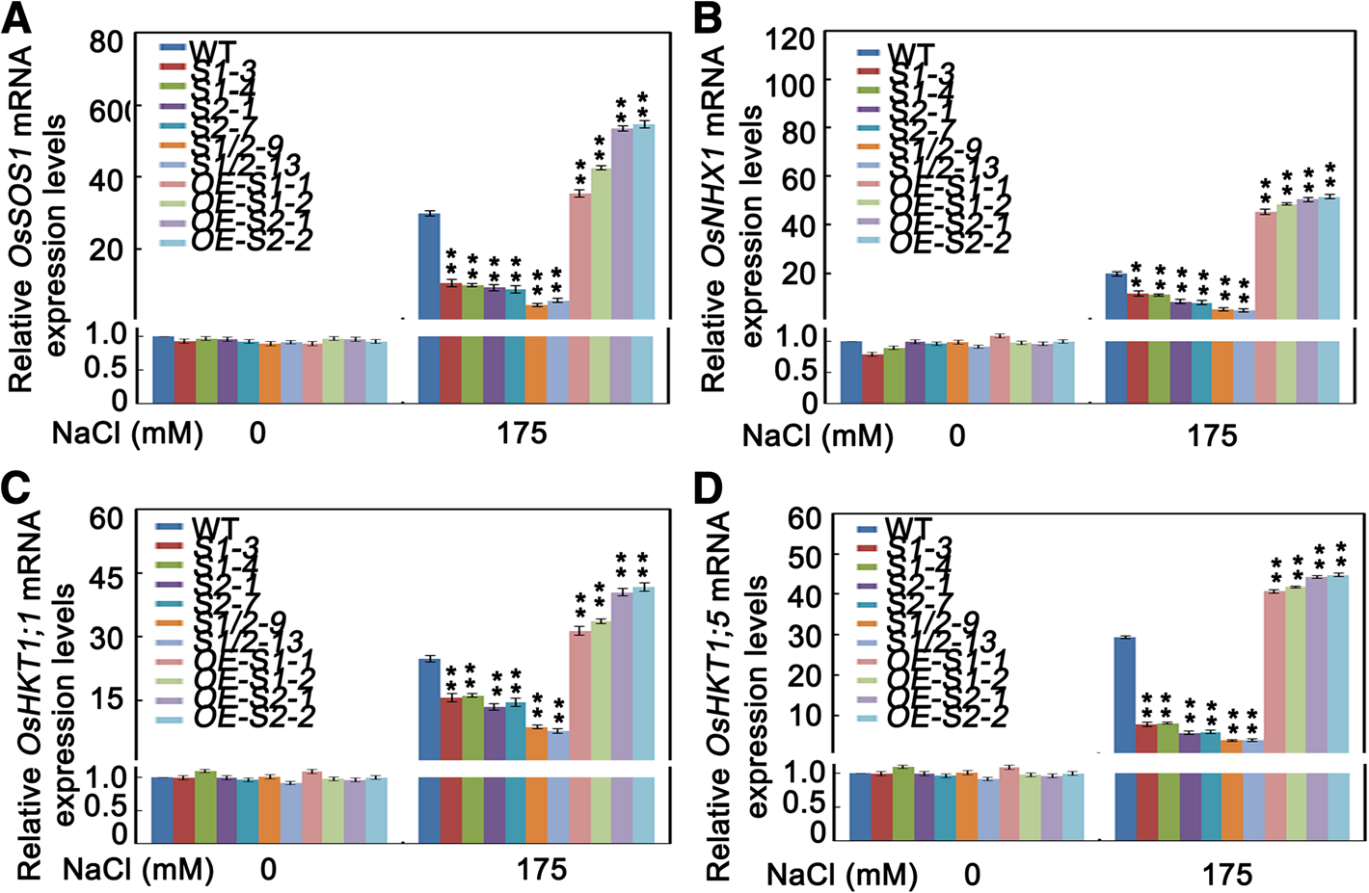
Expression of Na^+^ and K^+^ homeostasis genes. (**a**-**d**) Relative expression levels of *OsSOS1, OsNHX1, OsHKT1;1* and *OsHKT1;5* in the leaves of 4-week-old *sapk1*, *sapk2* and *sapk1/2* mutants; SAPK1-OE, SAPK2-OE plants and wild-type plants before and after salt stress. Values shown are means from three independent experiments. Error bars indicate the SD (n = 3). ** indicate statistically significant differences between mutant lines and wild type plants (P < 0.01)

A low Na^+^ content and low Na^+^/K^+^ ratio in the cytoplasm are essential to maintain ion homeostasis under both normal and salt stress conditions. To determine whether SAPK1 and SAPK2 are involved in regulating ion homeostasis under salt stress, we determined the Na^+^ and K^+^ contents in the *sapk1*, *sapk2* and *sapk1/*2 mutants, the *SAPK1-OE* and *SAPK2-OE* lines and in the wild-type plants. Rice plants were grown to 4 weeks and then treated with 175 mM NaCl for 5 d. Under normal conditions, the Na^+^ contents, K^+^ contents and the Na^+^/K^+^ ratios were similar between *sapk1*, *sapk2, sapk1/*2, *SAPK1-OE*, *SAPK2-OE* and the wild-type plants (Fig. 10a–f). Under salt stress, the K^+^ contents in the *sapk1* and *sapk2* mutants were significantly lower than that of the wild-type plants. The K^+^ contents in the *sapk1/*2 double mutants were much lower than those of the *sapk1* or *sapk2* single mutants (Fig. 9b). In contrast, the Na^+^ contents and Na^+^/K^+^ ratios in the *sapk1* and *sapk2* mutants were significantly higher than those of the wild-type plants, and the Na^+^ contents and Na^+^/K^+^ ratios in the *sapk1/*2 mutants were much higher than those of the *sapk1* or *sapk2* single mutants (Fig. 9a and c). In contrast, the *SAPK1-OE* and *SAPK2-OE* lines accumulated less Na^+^ and more K^+^, leading to a decreased Na^+^/K^+^ ratio in comparison with the wild type (Fig. 9d–f). Collectively, these observations suggest the possible roles of SAPK1 and SAPK2 in regulating Na^+^/K^+^ homeostasis under salt stress. Again, these roles appear to be collaborative.

To further investigate the molecular roles of SAPK1 and SAPK2 in Na^+^ homeostasis, we analyzed the expression of *OsSOS1*, *OsNHX1*, *OsHKT1;1* and *OsHKT1;5* in seedlings leaves from the mutant and overexpression lines. As shown in Fig. 10, under salt treatment, the expression levels of *OsSOS1*, *OsNHX1*, *OsHKT1;1* and *OsHKT1;5* were significantly reduced in the *sapk1*, *sapk2* and *sapk1/2* mutants. Expression levels of these genes were significantly increased in the *SAPK1-OE* and *SAPK2-OE* lines in comparison with the wild-type plants. These results show that SAPK1 and SAPK2 are involved in modulating the transcription of *OsSOS1*, *OsNHX1*, *OsHKT1;1* and *OsHKT1;5*.

## Discussion

SnRK2s are key molecules that unify different abiotic stress signals in plants [27]. Therefore, research on the function of the SnRK2s is meaningful to fully understand the mechanisms of abiotic stress responses in plants. Until now, no data on salt tolerance in SAPK gene knockouts in rice have been presented. To fully understand the roles of SAPK1 and SAPK2 in the salt-resistance mechanism, elucidation of their tissue specificities and analysis of mutants is crucial. To address these criteria, we generated the loss-of-function mutants such as *sapk1, sapk2* and *sapk1/2*, and the overexpression lines *SAPK1-OE* and *SAPK2-OE*.

In *Arabidopsis*, SnRK2.7 and SnRK2.8 are strongly activated by salt stress and weakly activated by ABA to regulate drought tolerance and plant growth [21, 25, 26]. *SnRK2.8* is expressed abundantly in roots and weakly in leaves, whereas *SnRK2.7* is expressed in roots, leaves and flowers [27]. SnRK2.8 and SnRK2.7 localize to the cytoplasm and nucleus [27]. SAPK1 and SAPK2 are rice SnRK2 II subfamily members and are homologous genes of SnRK2.7 and SnRK2.8 (Fig. 1). SAPK1 and SAPK2 are activated by salt treatment but not by ABA [17], suggesting that they may have important functions in plant salt responses. Our data showed that SAPK1 and SAPK2 expression were strongly induced by drought, NaCl, and PEG treatment, but not ABA (Fig. 2A). In addition, the highest *SAPK2* expression was in the leaves, followed by the roots; whereas *SAPK1* expression was highest in the roots, followed by the leaves (Fig. 2B). SAPK1 and SAPK2 were distributed in the nucleus and cytoplasm. Together, these results suggest that SAPK1 and SAPK2 may function in a different manner from SnRK2.7 and SnRK2.8.

Salt stress is particularly detrimental during germination and seedling growth. Slower growth is an adaptive feature for plant survival under salt stress because it allows plants to rely on multiple resources to combat stress [1]. Salt stress, like many other abiotic stresses, inhibits plant growth, resulting in a considerable reduction in the fresh weights of leaves, stems, and roots [35, 36]. Firstly, we characterized the sensitivity to salt stress of the *sapk1*, *sapk2* and *sapk1/*2 mutants during the germination and post-germination stages. All mutants, especially the *sapk1/*2 mutants, exhibited lower germination rates than the wild-type plants (Fig. 4a and b). In contrast, the *SAPK1-OE* and *SAPK2-OE* plants exhibited higher germination rates than those of the wild type (Fig. 5a and b). At the post germination stage, all mutants (especially the *sapk1/*2 mutants) exhibited reduced shoot and root lengths than the wild-type plants (Fig. 4c–e). The *SAPK1-OE* and *SAPK2-OE* plants exhibited longer shoot and root lengths than the wild-type plants (Fig. 5c–e). Next, we investigated the effects of SAPK1 and SAPK2 on growth at the seedling stage. Our phenotype analyses showed that, with salt treatment, the *sapk1*, *sapk2* and *sapk1/*2 mutants had more severe growth inhibition, developed chlorosis, and had reduced survival rates in comparison with the wild type (Fig. 6a–e). In contrast, the *SAPK1-OE* and *SAPK2-OE* plants had reduced sensitivity to salt and had higher survival rates in comparison with the wild type (Fig. 7a–e). Salt may affect plant growth indirectly by reducing photosynthesis [37]. The significantly lower chlorophyll contents in the *sapk1*, *sapk2* and *sapk1/2* mutants, and the higher chlorophyll contents in the *SAPK1-OE* and *SAPK2-OE* lines in comparison with the wild-type plants supports this hypothesis (Fig. 8a and b). Together, these results indicate that SAPK1 and SAPK2 increase rice survival rates by reducing growth inhibition and leaf chlorosis. Therefore, these proteins may function collaboratively as positive regulators of salt stress tolerance at the germination, post germination and seedling stages.

High concentrations of salt always induce osmotic stress [38]. The most immediate event following salt-induced osmotic stress is the loss of turgor pressure, resulting from changes in cell structure and membrane leakage [39]. Under salt stress, plants lower the osmotic potential by generating osmotically active metabolites such as proline, sugars, and sugar alcohols [40]. All osmolytes have the common characteristic of lowering the osmotic potential in the cytosolic compartment at relatively high concentrations without inhibiting metabolic reactions [39]. In the proline content analyses, we found that the proline contents under drought stress was significantly lower in the *sapk1, sapk2* and *sapk1/2* mutants, and much higher in the *SAPK1-OE* and *SAPK2-OE* lines than in wild-type plants (Fig. 8c and d). The *OsP5CS* expression levels were in accord with these results. These results suggest that SAPK1 and SAPK2 affect the generation of osmotically active metabolites such as proline. This response to salt stress at the seedling stage is predicted to reduce the osmotic stress and cell structure and membrane damage.

Salt tolerance in rice appears to correlate with the ability to scavenge reactive oxygen [41]. Salt stress also leads to increased ROS production. Plants cope with reactive oxygen stress by increasing the enzymatic and non-enzymatic scavenging activity of ROS [42, 43]. One of the non-enzymatic scavengers of reactive oxygen is ascorbic acid, while enzymatic scavengers include SOD and CAT. With salt treatment, the ascorbic acid contents and the SOD and CAT expression levels were significantly reduced in the *sapk1* and *sapk2* mutants, and especially in the *sapk1/2* mutants. These scavengers were significantly increased in the *SAPK1-OE* and *SAPK2-OE* lines in comparison with the wild-type plants (Fig. 8e–g). Moreover, the MDA contents and the relative electrolyte leakage levels were significantly higher in all mutants and were significantly lower in the *SAPK1-OE* and *SAPK2-OE* lines (Fig. 8c and d) in comparison with the wild type. MDA is a product of lipid peroxidation that arises from ROS activity, and is a stress-specific molecular marker that indicates the extent of cellular damage [34]. The most immediate event following salt-induced osmotic stress is the loss of turgor pressure, which due to changes in cell structure, different compositions of cell membranes and membrane leakage [44, 45]. Our results suggest that SAPK1 and SAPK2 protect plants from oxidative damage during salt stress by increasing the ascorbic acid content and SOD and CAT expression levels to enhance ROS detoxification.

In plants, K^+^ is an essential nutrient. K^+^ is also essential for enzyme activities and ionic homeostasis [46, 47]. Because Na^+^ inhibits many enzymes, it is important to prevent Na^+^ accumulating to a high level. When a high salt concentration is rapidly applied, NaCl shock leads to a rapid osmotic challenge, followed more slowly by accumulation of Na^+^. We analyzed the Na^+^ and K^+^ contents and the Na^+^/K^+^ ratio after a high salt concentration was applied. As shown in Fig. 9, the *sapk1*, *sapk2* and *sapk1/*2 mutants, especially the *sapk1/*2 mutants, accumulated more Na^+^ and less K^+^, leading to a higher Na^+^/K^+^ ratio in comparison with the wild type (Fig. 9a–c). In contrast, the *SAPK1-OE* lines and *SAPK2-OE* lines accumulated less Na^+^ and more K^+^, leading to a lower Na^+^/K^+^ ratio in comparison with the wild type (Fig. 9d–f). These observations suggest that SAPK1 and SAPK2 are probably involved in regulating Na^+^/K^+^ homeostasis under salt stress, and that their functions are collaborative.

Many of the components associated with the regulation of Na^+^ and K^+^ homeostasis have been characterized. Our qRT-PCR results showed that the expression levels of *OsSOS1*, *OsNHX1*, *OsHKT1;1* and *OsHKT1;5* were significantly reduced in the *sapk1*, *sapk2* and *sapk1/2* mutants, and were significantly increased in the *SAPK1-OE* lines and *SAPK2-OE* lines, in comparison with wild-type plants (Fig. 10a-d). The *OsSOS1* gene encodes a rice plasma membrane Na^+^/H^+^ exchanger that extrudes Na^+^ from the cortex cells at the root-soil interface, thereby reducing the net uptake of Na^+^ [48]. The reduced levels of *OsSOS1* transcript and the increased Na^+^ accumulation in the leaves of the *sapk1*, *sapk2* and *sapk1/2* mutants suggest that the decreased tolerance to salt in these plants is caused by extruding Na^+^ from the cortex cells. *OsNHX1* is essential for Na^+^ detoxification through Na^+^ sequestration into the vacuole [12, 49]. The reduced *OsNHX1* transcript levels in the *sapk1*, *sapk2* and *sapk1/2* mutants suggest that SAPK1 and SAPK2 affect the sequestration of Na^+^ in leaf cells. Members of the subfamily I HKT transporters are thought to mediate Na^+^ influx into root cells and to regulate the Na^+^ distribution between roots and shoots [15, 50–52]. *OsHKT1;1*, which is mainly expressed in the phloem of the leaf blades and was localized in plasma membrane in rice, plays an important role in reducing Na^+^ accumulation in shoots to cope with salt stress [53]. *OsHKT1;5* (*OsSKC1/HKT8*) participates in reabsorption of Na^+^ at the xylem parenchyma, thereby restricting the buildup of toxic concentrations of Na^+^ in photosynthetic tissues [54]. The reduced expression levels of *OsHKT1;1* and *OsHKT1;5* and increased Na^+^ accumulation in the leaves of the *sapk1*, *sapk2* and *sapk1/2* mutants suggests that the decreased tolerance to salt in these plants was caused by changes in the Na^+^ distribution between roots and shoots. Together, these results suggest that SAPK1 and SAPK2 may function collaboratively in reducing Na^+^ toxicity by affecting the Na^+^ distribution between roots and shoots, Na^+^ exclusion from the cell cytoplasm, and Na^+^ sequestration into the vacuoles. These processes may occur by SAPK1 and SAPK2 regulating the expression levels of the Na^+^- and K^+^- homeostasis related genes *OsSOS1*, *OsNHX1*, *OsHKT1;1* and *OsHKT1;5*.

## Conclusions

In this study, we have characterized the biochemical and physiological responses to salt stress of two rice subclass II SnRK2s, SAPK1 and SAPK2. To do this, we developed loss-of-function mutants (using the CRISPR/Cas9 system) and transgenic plants overexpressing SAPK1 and SAPK2. Expression profiling revealed that *SAPK1* and *SAPK2* expression were strongly induced by drought, NaCl, and PEG treatments, but not by ABA. In addition, the *SAPK2* expression level was highest in the leaves, followed by the roots; whereas the *SAPK1* expression level was highest in the roots, followed by the leaves. Both proteins were localized to the nucleus and cytoplasm. In phenotypic resistance analyses of salt stress, the *sapk1, sapk2* and *sapk1/2* mutants (especially the *sapk1/2* mutants) had reduced germination rates, increased growth inhibition, developed chlorosis, and had reduced survival rates in comparison with the wild type. In contrast, the *SAPK1-OE* and *SAPK2-OE* plants exhibited increased germination rates, reduced sensitivity, and increased survival in comparison with the wild type. These results suggest that SAPK1 and SAPK2 may function collaboratively as positive regulators of salt stress tolerance at the germination and seedling stages. Further study suggested that SAPK1 and SAPK2 affect osmotic stress and ROS detoxification in the salt stress response. SAPK1 and SAPK2 promote the generation of osmotically active metabolites such as proline, ROS scavengers such as ascorbic acid, as well as increased SOD and CAT expression. SAPK1 and SAPK2 may function collaboratively in reducing Na^+^ toxicity by affecting the Na^+^ distribution between the roots and shoots, excluding Na^+^ from the cytoplasm of cells, and sequestering Na^+^ into the vacuoles. These processes may occur via regulation of the expression of the Na^+^- and K^+^-homeostasis related genes *OsSOS1*, *OsNHX1*, *OsHKT1;1* and *OsHKT1;5*.

## Methods

### Plant growth conditions and stress treatment

Mutant lines of *sapk1, sapk2* and *sapk1/2*, overexpression lines of *SAPK1* and *SAPK2* and wide type plants were selected for the experimental works. All the experiments were performed using the seeds of same conditions. The sterilized seeds from different genotypes were germinated on half-strength Murashige and Skoog (MS) medium simultaneously and kept in growth chamber at 28 °C under long-day conditions (14 h light/10 h dark cycles). After 3 days germination, seedlings were transferred into half-strength liquid medium.

### Construction of mutants and overexpression plants

We employed the CRISPR/Cas9 system to generate *sapk1, sapk2* and *sapk1/2* mutants. The CRISPR/Cas9 plasmid was designed according to the protocol described previously [55]. Concisely, the third coding exons of *SAPK1*, *SAPK2* were selected for guide RNA design. The single target site was inserted between OsU3 and sgRNA in the pYLsgRNA-OsU3 vector, and then the OsU3-sgRNA cassette was cloned into the pMH-SA vector [55]. For the double editing of *SAPK1* and *SAPK2*, a tandem combination of OsU3-sgRNA (for *SAPK1*) and OsU6a-sgRNA (for *SAPK2*) was cloned into the pMH-SA vector.

For mutation detection, genomic DNA extracted from mutant seedlings (all plant) were used for PCR. Then PCR products were identified by comparing gRNA target sequences of *SAPK1* (atggaatatgctgctggtgg) and *SAPK2* (tagttatggaatatgctgc) to the rice reference genome [55]. In T_0_ generation, we collected 20 hygromycin-resistant plants for each gene. Based on mutation detection results, we identified two independent homozygous mutant lines for each gene in the T_1_ generation, which we named *sapk1–3, sapk1–4, sapk2–1, sapk2–7, sapk1/2–9* and *sapk1/2–13*. The primers used for CRISPR/Cas9 (U3-SAPK1-F, U3-SAPK1-R, U3-SAPK2-F and U3-SAPK2-R) and mutation detection (SAPK1-u3-F, SAPK2-u3-R, SAPK2-u3-F and SAPK2-u3-R) were listed in Additional file 1.

To generate the *SAPK1* and *SAPK2* overexpression transgenic plants, the full-length cDNA of *SAPK1* and *SAPK2* was cloned into the p1301 vector in the sense orientation behind the CaMV 35S promoter. Rice (*Oryza sativa* L. japonica.) was used for transformation.

### RNA extraction and qRT-PCR analysis

To detect the transcript levels of target genes under different stresses, wide type plants were grown in the same condition for 4 weeks. RNA extraction and qRT-PCR analysis followed the method of Lou et al. [32]. Gene-specific primers used in qRT-PCR analysis were listed in Additional file 1.

### GUS staining and subcellular localization of SAPK1 and SAPK2

For GUS reporter analysis, the putative promoters of *SAPK1* and *SAPK2* were amplified from genomic DNA using primers Promoter-SAPK1-F, Promoter-SAPK1-R and Promoter-SAPK2-F, Promoter-SAPK2-R (Additional file 1). The fused Pro_*SAPK1*_-GUS and Pro_*SAPK2*_-GUS was cloned into the p1300 vector. GUS staining was detected as described [32].

The full-length *SAPK1* and *SAPK2* CDS sequences were inserted into P30 to generate green fluorescent protein (GFP) fusion protein. The fusion constructs were employed for the transfection of Nicotiana benthamiana. GFP fluorescence was observed using a confocal laser scanning microscope (Olympus, http://www.olympus-global.com).

### Germination and early seedling growth assay

To test seed germination, seeds from different genotypes were surface-sterilized. Then sterilized seeds were planted on half-strength MS agar medium containing 0, 100, 150, 175, 200 mM NaCl. The germination rates were assessed at designated time. To test the growth performance at post-germination stage, the seeds of different genotypes were germinated on half-strength MS agar medium simultaneously. After 3 days, the seedlings were transferred to half-strength MS agar medium supplemented with 175 mM NaCl for further growth. Twenty seedlings were used to measure shoot and root length. All these experiments were repeated three times, with 30 seeds per sample.

### Salt tolerance assay

The sterilized seeds of different genotypes germinated on half-strength MS medium simultaneously for 3 days. Seedlings were then transplanted in half-strength liquid MS medium for 4 weeks. For NaCl tolerance assays, Seedlings were then transferred into half-strength MS liquid medium supplemented with 175 mM NaCl for 5 days (groups of SAPK1-OE and SAPK2-OE lines for 7 days). After treatment, the seedlings were transferred into fresh half-strength MS liquid medium to recover for 7 days.

### Analyses of relative electrolyte leakage and the content chlorophyll, proline, MDA and ascorbic acid

The 4-week-old seedlings of different genotypes treated with 175 mM NaCl for 5 days. Then relative ion leakage and the content chlorophyll, Proline, MDA and ascorbic acid were analyzed.

The relative electrolyte leakage was checked following the method of Lou et al. [32]. The conductance of H_2_O was measured by conductivity meter (HORIBA TWIN COND B-173).

Chlorophyll was extracted with 80% acetone from leaves of salt-treated plants. Chlorophyll content was determined at 663 nm and 645 nm according to the method described by Lichtenthaler [56].

The content of free proline in leaves was estimated as described [32]. And the absorbance at 520 nm was measured by spectrophotometer. L-Pro was used as a standard to calculate the proline concentration.

The content of MDA in leaves was detected as described [32]. And the absorbance at 532 nm was measured by spectrophotometer. The MDA content was expressed as nmol g^− 1^ FW.

The content of ascorbic acid was detected using commercial assay kits purchased from Nanjing Jiancheng Bioengineering Institute (Nanjing, China). And the absorbance at 536 nm was measured.

### Ion content determination

Ion content in rice leaves was measured as described previously [57] with minor modifications. Briefly, 4-weeks-old plants were treated with 175 mM NaCl and allowed to grow for 5 days. Leaves were harvested. Then ion content was determined using an inductively coupled plasma optical emission spectrometer (ICP-OES) (SPS3100, SII Nano Technology Inc., Japan).

### Statistical analysis

For the above assays, consistent results were obtained from at least three experiments, and the result from one experimental was exhibited here. Average of three replicates was calculated to represent each data point. Excel 2010 was used for making charts. Significant difference among different treatments was identified by an analysis of variance by using SigmaPlot10.0. The data represent mean ± standard error (SE) of three independent experiments.

## Additional file

Additional file 1: Primers and oligos used in this study. Primers and oligos used for plasmid constrcutions, mutation detection and *qRT-PCR*. (DOCX 17 kb)

## Abbreviations

ABA: abscisic acid
CAT: catalase
GFP: green fluorescent protein
GUS: β-glucuronidase
HKTs: high-affinity K^+^ transporters
MDA: malondialdehyde
MS: Murashige and Skoog
NHXs: tonoplast-localized Na^+^/H^+^ or K^+^/H^+^ exchangers
qRT-PCR: quantitative reverse transcription-PCR
ROS: reactive oxygen species
SAPK: osmotic stress/ABA-activated protein kinase
SnRK2: the sucrose non-fermenting-1-related protein kinase 2
SOD: superoxide dismutase
SOSs: the plasma membrane Na^+^/H^+^ antiporters salt overly sensitive
