# Supplementary material for: The sucrose non-fermenting-1-related protein kinases SAPK1 and SAPK2 function collaboratively as positive regulators of salt stress tolerance in rice
Source: BMC Plant Biol. 2018 Sep 20;18:203. doi: 10.1186/s12870-018-1408-0 (PMC6146518; doi:10.1186/s12870-018-1408-0)
Supplement: Supplementary file 1 — Primers and oligos used in this study. Primers and oligos used for plasmid constrcutions, mutation detection and qRT-PCR. (DOCX 17 kb) [file 12870_2018_1408_MOESM1_ESM.docx]

**Additional file 1. Primers and oligos used in this study**

| Name | Primer sequence (5’-3’) |
| --- | --- |
| **Plasmid Constrcutions and Mutation Detection** | |
| U3-SAPK1-F | ggcaatggaatatgctgctggtgg |
| U3-SAPK1-R | ccaccagcagcatattccatcaaa |
| SAPK1-u3-F | TTGAATACAACAAGTTGGCCCA |
| SAPK1-u3-R | GTGTGACACTGCCATCCAAGAG |
| Promoter-SAPK1-F | TAAAGCTTTGTTAGGAATTTTTACGATTGGCCTTGC |
| Promoter-SAPK1-R | ATAGGATCCATCCCCTCAACGACCTCGCTCC |
| P30-SAPK1-F | ATAGAGCTCATGGAGCGGTACGAGGTGATGAGGG |
| P30-SAPK1-R | ATAGGATCCCAAGGCGCACACGAAGTCCCC |
| P1301-SAPK1-F | ATAGGATCCATGGAGCGGTACGAGGTGATGAGGG |
| P1301-SAPK1-R | ATAGGTACCTCACAAGGCGCACACGAAGTCCCC |
| U3-SAPK2-F | GGCATAGTTATGGAATATGCTGC |
| U3-SAPK2-R | ATCAATACCTTATACGACGCAAA |
| SAPK2-u3-F | GGAAATGAAAGTGACCCAGCAG |
| SAPK2-u3-R | TGCTCCCATCCAAGAGAGTATTTT |
| Promoter-SAPK2-F | ATAGTCGACAAGATCCTGGGATCAAAGAAAGCTTCG |
| Promoter-SAPK2-R | ATAGGATCCCCCCACCTCCCACGACAACTCC |
| P30-SAPK2-F | ATAGGATCCATGGAGAGGTACGAGGTGATCAAGGACAT |
| P30-SAPK2-R | ATATCTAGACAATGCGCACACGAAGTCGC |
| P1301-SAPK2-F | ATAGGATCCATGGAGAGGTACGAGGTGATCAAGGACAT |
| P1301-SAPK2-R | ATAGGTACCTCACAATGCGCACACGAAGTCGC |
| ***qRT-PCR*** | |
| OsActin-RT-F | AGCTGCGGGTATCCATGAGA |
| OsActin-RT-R | GCAATGCCAGGGAACATAGTG |
| RT-OsP5CS1-F | GCTGACATGGATATGGCAAAAC |
| RT-OsP5CS1-R | GTAAGGTCTCCATTGCATTGCA |
| RT-OsSOS1-F | CATGAAGTCCGGTCAGGGTC |
| RT-OsSOS1-R | GACTGCACAGACAGCAGGG |
| RT-OsNHX1-F | CCAAAGTCCCTGCATTCTCCT |
| RT-OsNHX1-R | GCGCCAGTAGTAGTGGACAG |
| RT-OsHKT1;5-F | CGTCGAGGTTATCAGTGCGT |
| RT-OsHKT1;5-R | GCTTCCCTTGTTTGCTCCAC |
| RT-OsHKT1;1-F | ATGTGGGGTTCTCAATGGGC |
| RT-OsHKT1;1-R | ATCAGTTTGCCAGAGTCGCT |
| RT-OsCAT -F | TACTTCCCATCCCGCTACGA |
| RT-OsCAT-R | TCCTTACATGCTCGGCTTCG |
| RT-OsCu/Zn-SOD1-F | CAGGTTGAGGGAGTCGTCAC |
| RT-OsCu/Zn-SOD1-R | GGTTGCCTCAG CTACACCTT |
